# The future of International Classification of Diseases coding in steatotic liver disease: An expert panel Delphi consensus statement

**DOI:** 10.1097/HC9.0000000000000386

**Published:** 2024-02-03

**Authors:** Hannes Hagström, Leon A. Adams, Alina M. Allen, Christopher D. Byrne, Yoosoo Chang, Ajay Duseja, Henning Grønbæk, Mona H. Ismail, Peter Jepsen, Fasiha Kanwal, Jennifer Kramer, Rohit Loomba, Henry E. Mark, Philip N. Newsome, Mary E. Rinella, Ian A. Rowe, Seungho Ryu, Arun Sanyal, Jörn M. Schattenberg, Marina Serper, Nick Sheron, Tracey G. Simon, C. Wendy Spearman, Elliot B. Tapper, Marcela Villota-Rivas, Sarah H. Wild, Vincent Wai-Sun Wong, Yusuf Yilmaz, Shira Zelber-Sagi, Fredrik Åberg, Jeffrey V. Lazarus

**Affiliations:** 1 Department of Upper GI, Division of Hepatology, Karolinska University Hospital, Stockholm, Sweden; 2 Department of Medicine, Huddinge, Karolinska Institutet, Stockholm, Sweden; 3 Medical School, the University of Western Australia, Perth, Australia; 4 Department of Medicine, Division of Gastroenterology and Hepatology, Mayo Clinic, Rochester, Minnesota, USA; 5 Nutrition and Metabolism, Faculty of Medicine, University of Southampton, Southampton, UK; 6 Southampton National Institute for Health Research Biomedical Research Centre, University Hospital Southampton, Southampton General Hospital, Southampton, UK; 7 Center for Cohort Studies, Total Healthcare Center, Kangbuk Samsung Hospital, Sungkyunkwan University School of Medicine, Seoul, Republic of Korea; 8 Department of Hepatology, Postgraduate Institute of Medical Education and Research, Chandigarh, India; 9 Department of Hepatology and Gastroenterology, Aarhus University Hospital, Aarhus, Denmark; 10 Department of Internal Medicine, Division of Gastroenterology, King Fahd Hospital of the University, Al-Khobar, Saudi Arabia; 11 College of Medicine, Imam Abdulrahman Bin Faisal University, Dammam, Saudi Arabia; 12 Baylor College of Medicine and Michael E. DeBakey VA Medical Center, Houston, Texas, USA; 13 NAFLD Research Center, Division of Gastroenterology and Epidemiology, University of California San Diego, La Jolla, California, USA; 14 Independent consultant, Nottingham, UK; 15 National Institute for Health Research Birmingham Biomedical Research Centre at University Hospitals Birmingham NHS Foundation Trust and the University of Birmingham, Birmingham, UK; 16 Centre for Liver and Gastrointestinal Research, Institute of Immunology and Immunotherapy, University of Birmingham, Birmingham, UK; 17 Department of Medicine, University of Chicago, Chicago, Illinois, USA; 18 Leeds Institute for Medical Research, University of Leeds, Leeds, UK; 19 Department of Clinical Research Design and Evaluation, SAIHST, Sungkyunkwan University, Seoul, Republic of Korea; 20 Department of Internal Medicine, Stravitz-Sanyal Institute of Liver Disease and Metabolic Health, VCU School of Medicine and Health System and Division of Gastroenterology, VCU School of Medicine, Richmond, Virginia, USA; 21 Department of Internal Medicine II, University Hospital of Saarland, Homburg, Germany; 22 Department of Medicine, Division of Gastroenterology and Hepatology, University of Pennsylvania Perelman School of Medicine, Philadelphia, Pennsylvania, USA; 23 The Roger Williams Institute of Hepatology, Foundation for Liver Research, Kings College London; 24 Division of Gastroenterology and Hepatology, Massachusetts General Hospital, Boston, Massachusetts, USA; 25 Harvard Medical School, Boston, Massachusetts, USA; 26 Department of Medicine, Division of Hepatology, Faculty of Health Sciences, University of Cape Town, Cape Town, South Africa; 27 Division of Gastroenterology and Hepatology, University of Michigan, Ann Arbor, Michigan, USA; 28 Barcelona Institute for Global Health (ISGlobal), Hospital Clínic, University of Barcelona, Barcelona, Spain; 29 Usher Institute, University of Edinburgh, Edinburgh, UK; 30 Department of Medicine and Therapeutics, The Chinese University of Hong Kong, Hong Kong; 31 Department of Gastroenterology, School of Medicine, Recep Tayyip Erdoğan University, Rize, Turkey; 32 School of Public Health, Faculty of Social Welfare and Health Sciences, University of Haifa, Haifa, Israel; 33 Department of Gastroenterology, Tel Aviv Medical Centre, Tel Aviv, Israel; 34 Transplantation and Liver Surgery, Helsinki University Hospital, Helsinki, Finland; 35 University of Helsinki, Helsinki, Finland; 36 CUNY Graduate School of Public Health and Health Policy (CUNY SPH), New York, New York, USA; 37 Faculty of Medicine and Health Sciences, University of Barcelona, Barcelona, Spain

## Abstract

**Background:**

Following the adoption of new nomenclature for steatotic liver disease, we aimed to build consensus on the use of International Classification of Diseases codes and recommendations for future research and advocacy.

**Methods:**

Through a two-stage Delphi process, a core group (n = 20) reviewed draft statements and recommendations (n = 6), indicating levels of agreement. Following revisions, this process was repeated with a large expert panel (n = 243) from 73 countries.

**Results:**

Consensus ranged from 88.8% to 96.9% (mean = 92.3%).

**Conclusions:**

This global consensus statement provides guidance on harmonizing the International Classification of Diseases coding for steatotic liver disease and future directions to advance the field.

## INTRODUCTION

Nomenclature changes in the field of steatotic liver disease (SLD) were recently proposed and are currently being adopted by a wide range of stakeholders.^1^ Among the suggested modifications, the change from NAFLD to metabolic dysfunction–associated steatotic liver disease (MASLD) reflects a drop of the “nonalcoholic” label, enabling the inclusion of positive diagnostic criteria while removing potentially stigmatizing classification. The intake of alcohol as a disease contributor is also acknowledged in the new nomenclature, with the introduction of the term “MASLD and alcohol-associated liver disease (ALD)”, abbreviated as MASLD and ALD (MetALD).^1^ Moreover, the nomenclature process introduced new defining criteria for MASLD and MetALD. Whereas studies have demonstrated almost complete overlap between populations defined by NAFLD and MASLD criteria, the cutoffs for alcohol use in the MetALD definition remain to be clarified by future studies.^2–4^

As a consequence of these nomenclature changes and to aid in their implementation, administrative coding will need to be adjusted. Globally, the International Classification of Diseases (ICD) coding system is the most used. We thus aimed to build consensus on the appropriateness of using current ICD NAFLD and NASH codes to code MASLD and metabolic dysfunction–associated steatohepatitis (MASH), respectively. We also sought to develop recommendations to guide research and advocacy on amending future ICD codes for SLD. While ICD systems vary at the local level (eg, which version is in use), ICD-10 is currently the dominant system. Nonetheless, following its release in 2022, ICD-11 use will be gradually introduced over the coming years.

## METHODS

We performed a two-stage Delphi process whereby, first, a core group of people (n = 20) indicated their agreement or disagreement with statements and recommendations (n = 6) (Supplemental Table 1, http://links.lww.com/HC9/A809) using “yes” to agree and “no” to disagree, through Microsoft Forms, from July 23 to August 6, 2023. Respondents were also invited to provide qualitative feedback on each item and overall, which was considered during item revisions. This group included individuals who had previously contributed to a consensus statement on the use of NAFLD ICD codes in research^5^ and key opinion leaders involved in the nomenclature change.

The second stage involved inviting a panel of individuals with SLD experience to indicate their level of agreement (“agree,” “somewhat agree,” “somewhat disagree,” or “disagree”) with the modified items (n=6) (Table 1), using the described methodology,^6^ through Qualtrics XM, from October 6–23, 2023. Respondents were also invited to provide qualitative feedback on each item and overall, which was considered during manuscript writing. Invitees who were not familiar with ICD codes and their use could opt out. Respondents who did not feel qualified to indicate their level of agreement with a survey item could choose the option “not qualified to respond.” For the purposes of this study, we defined reaching consensus as having > 80% agreement on each item, with overall agreement being the sum of the “agree” and “somewhat agree” categories in stage 2.

**TABLE 1:** Final consensus statements and recommendations

Statements		Grade	A (%)	SA (%)	A+SA (%)	SD (%)	D (%)	NQ (%)	N
1	MASLD is currently best coded using the ICD-10 code for NAFLD (K76.0).	B	67.4	21.5	88.8	5.6	5.6	4.1	233
2	MASH is currently best coded using the ICD-10 coding for NASH (K75.8 or K75.81, depending on the setting).	A	70.8	21.0	91.8	4.3	3.9	4.1	233
3	ALD is best coded using the “alcohol-associated liver disease” spectrum of ICD-10 codes (K70).	A	84.6	12.3	96.9	2.2	0.9	6.6	227
4	As no appropriate MetALD ICD-10 code exists, clinical research and health care professionals should use ICD coding for the more relevant part of MASLD/ALD on an individual basis while awaiting ICD-10/11 definition changes by the World Health Organization.	B	62.3	26.8	89.2	6.9	3.9	4.9	231
Recommendations									
5	Research should focus on identifying how to best distinguish between MASLD, MetALD, and ALD when using historical data sources (eg, register-based data).	A	68.5	22.7	91.2	5.9	2.9	2.1	238
6	International societies should advocate for a global update of ICD terminology by the World Health Organization to better reflect the nomenclature change, including separate diagnostic codes for MASLD, MASH, MetALD, ALD, and cryptogenic steatotic liver disease.	A	86.4	9.5	95.9	2.9	1.2	0.4	242
Mean % agreement		73.3	19.0	92.3	—	—	—	—	—

*Notes:* Percentages may add up to more than 100 due to rounding. Grades are based on the percentage of combined agreement (agree+somewhat agree): A, 90%–99% combined agreement; B, 78%–89% combined agreement. Responses to each statement and recommendation are presented as percentages of the total responses.

Abbreviations: A, agree; ALD, alcohol-associated liver disease; D, disagree; ICD, International Classification of Diseases; MASH, metabolic dysfunction-associated steatohepatitis; MASLD, metabolic dysfunction-associated steatotic liver disease; MetALD, MASLD and ALD; N, total number of responses; NQ, percentage of participants that indicated that they were not qualified to respond; SA, somewhat agree; SD, somewhat disagree.

### Ethical considerations

This study received ethical review exemption from the Hospital Clínic of Barcelona, Spain, ethics committee on October 4, 2023. All research was conducted in accordance with the Declarations of Helsinki and Istanbul. Respondents consented to participating, and data were anonymized for all analyses.

## RESULTS

A total of 479 individuals were invited to participate in stage 2, of whom 269 (56.2%) responded. Of these, 26 (9.7%) opted out as they were not familiar with ICD codes and their use. The 243 respondents (90.3%) who completed the survey worked in 73 countries and had a mean age of 53.9 (SD: 9.4). Most respondents were male (65.4%), worked in high-income countries (66.3%) and in the Europe and Central Asia World Bank region (41.2%), and primarily worked in academia (67.9%) and as clinicians/medical doctors (72.8%) (Supplemental Table 2, http://links.lww.com/HC9/A809, contains further panelist details).

In stage 2, consensus ranged from 88.8% to 96.9% (mean = 92.3%). Four items had < 80% “agree” responses and relied more heavily on the “somewhat agree” category to reach a consensus. A total of 351 qualitative comments were provided across items. There was ≥ 88.8% consensus that MASLD, MASH, and ALD are currently best coded with K76.0, K75.8, or K75.81, and the K70 spectrum of ICD-10 codes, respectively. As for MetALD, which has no ICD code as it was newly introduced, 89.2% agreed that using ICD coding for the perceived dominant disease driver (MASLD or ALD) on an individual basis was preferable while awaiting updates to the ICD system. In terms of recommendations, 91.2% of participants agreed that research should prioritize how best to distinguish between MASLD, MetALD, and ALD when using historical data. Furthermore, the consensus that international societies should advocate for a global update of ICD terminology to better reflect the SLD nomenclature changes was 86.4%.

## DISCUSSION

This study found that, among a large panel of experts working across 73 counties, there was a high degree of consensus that NAFLD and NASH ICD codes can be updated to reflect the new MASLD and MASH names and definitions, respectively, without the need for new codes. Renaming the administrative terms across various systems and countries to reflect the nomenclature change should be a priority. This is important, as introducing coding changes may lead to considerable difficulties in comparing study results and interpreting disease epidemiology patterns across settings and over time. It should be noted that definition and ICD code modifications will not mitigate the challenge of correctly calculating the amount of alcohol consumed by patients, but we hope that the recommendation of focusing research on identifying how to best distinguish between MASLD, MetALD, and ALD will promote investigations around this topic. Further work to introduce novel ICD codes to specifically define MetALD is needed, which may be achieved through discussions with national and regional norm-setting bodies and the World Health Organization, which maintains and updates the ICD system.

## CONCLUSIONS

This global expert consensus statement recommends that the currently available ICD codes for NAFLD and NASH can be used to define MASLD and MASH, respectively, although advocacy is needed to update ICD terminology to better reflect the nomenclature change and introduce new codes for MetALD specifically.

## Supplementary Material
