# Supplementary material for: The future of International Classification of Diseases coding in steatotic liver disease: An expert panel Delphi consensus statement
Source: Hepatol Commun. 2024 Feb 3;8(2):e0386. doi: 10.1097/HC9.0000000000000386 (PMC10843430; doi:10.1097/HC9.0000000000000386)
Supplement: Supplementary file 1 [file hc9-8-e0386-s001.docx]

**Supplementary Table 1**. Initial and revised consensus statements and recommendations.

| **Initial statements^a^** | **Grade** | **Agree (%)** | **General revision input** | **Revised statements^b,c^** |
| --- | --- | --- | --- | --- |
| 1. MASLD is best coded using the current ICD-10 code for NAFLD (K76.0). | A | 90 | Keep as is for now. Consider changes to require including cardiometabolic risk factors in upcoming updates of ICD coding. | 1. MASLD is **currently** best coded using the current ICD-10 code for NAFLD (K76.0). |
| 2. MASH is best coded using the current ICD-10 code for NASH (K75.8). | A | 85 | Some countries or settings use, e.g., K75.81, but it is not available everywhere. A specific code for MASH is desired. | 2. MASH is **currently** best coded using the ICD-10 **coding** for NASH (K75.8 **or K75.81, depending on the setting**). |
| 3. Alcohol-related liver disease is best coded using the current ICD-10 codes under the spectrum of “Alcoholic liver disease” (K70). | A | 85 | No code exists for MetALD. | 3. **ALD** is best coded using the “**a**lcoholic liver disease” spectrum of ICD-10 codes (K70). |
| 4. At this time, no appropriate ICD-10 code exists for MetALD. Awaiting any changes in the ICD-10/11 definitions by WHO, clinicians should use coding for the more relevant part of MASLD/ALD on an individual basis. | A | 85 | Try to implement a new code in ICD-11 for MetALD. | 4. **As** no appropriate MetALD ICD-10 code exists**,** **clinical research and healthcare professionals** should use ICD coding for the more relevant part of MASLD/ALD on an individual basis, **while** awaiting ICD-10/11 definition changes by the **World Health Organization**. |
| 5. Use of coding for MASLD/MASH and ALD in the same patient should be discouraged. | NA^d^ | 60 | Difficult in the absence of a MetALD ICD-code. | Statement not presented to the panel as consensus was not reached. |
| **Initial recommendations^a^** | **Grade** | **Agree (%)** | **General revision input** | **Revised recommendations^b,c^** |
| 6. Future research should prioritize studies on how to best distinguish between MASLD, MetALD, and ALD when using register-based data sources. | A | 95 | Prioritize over what? Use of historical data - how about clinical practice? | 5. Research should **focus** on **identifying** how to best distinguish between MASLD, MetALD, and ALD when using **historical data** sources **(e.g.,** register-based data**)**. |
| - | - | - | - | 6. International societies should advocate for a global update of ICD terminology by the World Health Organization to better reflect the nomenclature change, including separate diagnostic codes for MASLD, MASH, MetALD, ALD, and cryptogenic steatotic liver disease.^e^ |

*Abbreviations:* ALD, alcohol-related liver disease; ICD, International Classification of Diseases; MASH, metabolic dysfunction-associated steatohepatitis; MASLD, metabolic dysfunction-associated steatotic liver disease; MetALD, MASLD and ALD; NA, not applicable; NAFLD, nonalcoholic fatty liver disease; NASH, nonalcoholic steatohepatitis; WHO, World Health Organization.

Grades are based on the percentage of agreement: A, 90-99% agreement; B, 78-89% agreement. Responses to each statement and recommendation are presented as percentages of the total responses.

^a^Statements and recommendations presented to core group. ^b^Revised statements and recommendations post core group input, which were then presented to the panel. ^c^Revisions denoted in bold. ^d^Grading not applicable if an item has <67% combined agreement. ^e^Recommendation added as suggested by core group upon revisions.

**Supplementary Table 2.** Delphi panel characteristics (n=243).

| **Characteristic** | **n (%)** |  |
| --- | --- | --- |
| **Gender** | |  |
| Woman | 82 (33.7) |  |
| Man | 159 (65.4) |  |
| Non-binary or gender diverse | 1 (0.4) |  |
| Prefer not to say | 1 (0.4) |  |
| **Age, mean [SD]** | |  |
| All | 53.9 [9.4] |  |
| **Country of birth (n=81), by income level** | |  |
| Low or middle | 99 (40.7) |  |
| High | 144 (59.3) |  |
| **Global region^a^ of birth** | |  |
| East Asia and Pacific | 29 (11.9) |  |
| Europe and Central Asia**^b^** | 105 (43.2) |  |
| Latin America and Caribbean | 38 (15.6) |  |
| Middle East and North Africa | 18 (7.4) |  |
| North America | 29 (11.9) |  |
| South Asia | 13 (5.3) |  |
| Sub-Saharan Africa | 11 (4.5) |  |
| **Country of work (n=73), by income level** | |  |
| Low or middle | 82 (33.7) |  |
| High | 161 (66.3) |  |
| **Global region^a^ of work** | |  |
| East Asia and Pacific | 28 (11.5) |  |
| Europe and Central Asia**^c^** | 100 (41.2) |  |
| Latin America and Caribbean | 33 (13.6) |  |
| Middle East and North Africa | 15 (6.2) |  |
| North America | 51 (21.0) |  |
| South Asia | 8 (3.3) |  |
| Sub-Saharan Africa | 8 (3.3) |  |
| **Employment status** | |  |
| Employed | 233 (95.9) |  |
| Unemployed | 3 (1.2) |  |
| Retired | 7 (2.9) |  |
| **Sectors worked in^d^** | |  |
| Academia | 206 (84.4) |  |
| Public | 109 (44.9) |  |
| Private | 60 (24.7) |  |
| Civil society | 5 (2.1) |  |
| **Sector primarily worked in** | |  |
| Academia | 165 (67.9) |  |
| Public | 57 (23.5) |  |
| Private | 19 (7.8) |  |
| Civil society | 2 (0.8) |  |
| **Fields worked in^d^** | |  |
| Clinician/medical doctor | 219 (90.1) |  |
| Allied health professional | 4 (1.6) |  |
| Healthcare administration | 20 (8.2) |  |
| Clinical research | 155 (63.8) |  |
| Non-clinical research | 45 (18.5) |  |
| Patient advocacy | 15 (6.2) |  |
| Policy | 16 (6.6) |  |
| Education/pedagogy | 57 (23.5) |  |
| Other | 6 (1.6) |  |
| **Field primarily worked in** | |  |
| Clinician/medical doctor | 177 (72.8) |  |
| Allied health professional | 2 (0.8) |  |
| Healthcare administration | 1 (0.4) |  |
| Clinical research | 41 (16.9) |  |
| Non-clinical research | 12 (4.9) |  |
| Patient advocacy | 2 (0.8) |  |
| Policy | 1 (0.4) |  |
| Education/pedagogy | 5 (2.1) |  |
| Other | 2 (0.8) |  |
| **Years working in steatotic liver disease field** | |  |
| 1 to 11 | 77 (31.7) |  |
| 12 to 22 | 117 (48.1) |  |
| 23 to 33 | 44 (18.1) |  |
| 34 to 44 | 3 (1.2) |  |
| >44 | 2 (0.8) |  |
| **Publications authored focused on steatotic liver disease** | |  |
| <6 | 56 (23.0) |  |
| 6 to 25 | 75 (30.9) |  |
| 26 to 50 | 30 (12.3) |  |
| 51 to 100 | 39 (16.0) |  |
| >101 | 43 (17.7) |  |
| **Frequency of ICD code use in clinical work (n=236)^e^** | |  |
| Daily | 108 (45.8) |  |
| Weekly | 57 (24.2) |  |
| Monthly | 10 (4.2) |  |
| Less than monthly | 21 (8.9) |  |
| No ICD code use in clinical work | 40 (16.9) |  |
| **Experience in doing research using ICD codes (n=161)^f^** | |  |
| Yes | 98 (60.9) |  |
| No | 63 (39.1) |  |
| **Regional liver association membership^d^** | |  |
| AASLD | 120 (49.4) |  |
| APASL | 29 (11.9) |  |
| ALEH | 32 (13.2) |  |
| EASL | 140 (57.6) |  |
| No membership | 48 (19.8) |  |
| **Regional liver association primarily associated with (n=195)^g^** | |  |
| AASLD | 53 (27.2) |  |
| APASL | 25 (12.8) |  |
| ALEH | 26 (13.3) |  |
| EASL | 91 (46.7) |  |
| **Area of national professional association/society membership^d^** | |  |
| Liver disease/hepatology | 192 (79.0) |  |
| Gastrointestinal disease/gastroenterology | 133 (54.7) |  |
| Obesity | 27 (11.1) |  |
| Nutrition | 14 (5.8) |  |
| Diabetes/endocrinology | 35 (14.4) |  |
| Heart disease/cardiology | 4 (1.6) |  |
| Cancer/oncology | 7 (2.9) |  |
| Primary care | 13 (5.3) |  |
| Internal medicine | 6 (2.5) |  |
| Public health | 3 (1.2) |  |
| Transplantation | 3 (1.2) |  |
| Other | 10 (4.1) |  |
| No membership | 7 (2.9) |  |

*Abbreviations:* AASLD, American Association for the Study of Liver Diseases; ALEH, Asociación Latinoamericana para el Estudio del Hígado (Latin American Association for the Study of the Liver); APASL, Asian Pacific Association for the Study of the Liver; EASL, European Association for the Study of the Liver; ICD, International Classification of Diseases.

Percentages may add up to more than 100 due to rounding.

^a^Based on World Bank regions. ^b^n=4 participants were born in Central Asia. ^c^n=4 participants work in Central Asia. ^d^Sum may exceed sample size as participants could choose >1 response. ^e^Question only posed to respondents who chose clinician/medical doctor, allied health professional, clinical research, or healthcare administration as fields worked in. ^f^Question only posed to respondents who chose clinical research or non-clinical research as fields worked in. ^g^n only includes respondents who are members of any of the regional liver associations listed.
